# Proposal of selective wedge instillation of pulmonary surfactant for COVID-19 pneumonia based on computational fluid dynamics simulation

**DOI:** 10.1186/s12890-021-01435-4

**Published:** 2021-02-22

**Authors:** Hiroko Kitaoka, Hisato Kobayashi, Takayuki Takimoto, Takashi Kijima

**Affiliations:** 1 Department of Biomedical Engineering, Tokyo University of Agriculture and Technology, Koganei, Japan; 2 Department of Pediatrics, Keio University School of Medicine, Tokyo, Japan; 3 Department of Internal Medicine, National Hospital Organization Kinki-Chuo Chest Medical Center, Sakai, Japan; 4 Department of Respiratory Medicine and Hematology, Hyogo College of Medicine, Nishinomiya, Japan; 5 Department of Biomedical Engineering, Tokyo University of Agriculture and Technology, 2-24-16 Nakamachi, Koganei-shi, Tokyo 184-8588 Japan

**Keywords:** Alveolar collapse, Diffuse alveolar damage, ARDS, Interstitial pneumonia, Type II pneumocyte, Wedge pressure, Bronchoscopic therapy

## Abstract

**Background:**

The most important target cell of SARS-CoV-2 is Type II pneumocyte which produces and secretes pulmonary surfactant (PS) that prevents alveolar collapse. PS instillation therapy is dramatically effective for infant respiratory distress syndrome but has been clinically ineffective for ARDS. Nowadays, ARDS is regarded as non-cardiogenic pulmonary edema with vascular hyper-permeability regardless of direct relation to PS dysfunction. However, there is a possibility that this ineffectiveness of PS instillation for ARDS is caused by insufficient delivery. Then, we performed PS instillation simulation with realistic human airway models by the use of computational fluid dynamics, and investigated how instilled PS would move in the liquid layer covering the airway wall and reach to alveolar regions.

**Methods:**

Two types of 3D human airway models were prepared: one was from the trachea to the lobular bronchi and the other was from a subsegmental bronchus to respiratory bronchioles. The thickness of the liquid layer covering the airway was assigned as 14 % of the inner radius of the airway segment. The liquid layer was assumed to be replaced by an instilled PS. The flow rate of the instilled PS was assigned a constant value, which was determined by the total amount and instillation time in clinical use. The PS concentration of the liquid layer during instillation was computed by solving the advective-diffusion equation.

**Results:**

The driving pressure from the trachea to respiratory bronchioles was calculated at 317 cmH_2_O, which is about 20 times of a standard value in conventional PS instillation method where the driving pressure was given by difference between inspiratory and end-expiratory pressures of a ventilator. It means that almost all PS does not reach the alveolar regions but moves to and fro within the airway according to the change in ventilator pressure. The driving pressure from subsegmental bronchus was calculated at 273 cm H_2_O, that is clinically possible by wedge instillation under bronchoscopic observation.

**Conclusions:**

The simulation study has revealed that selective wedge instillation under bronchoscopic observation should be tried for COVID-19 pneumonia before the onset of ARDS. It will be also useful for preventing secondary lung fibrosis.

## Background

It is known that SARS-CoV-2 binds angiotensin converting enzyme 2 (ACE2) on the pulmonary epithelial cells and causes acute interstitial pneumonia. ACE2 and TMPRSS2 (Type II transmembrane serine protease) are necessary for invasion into a cell; the two enzymes coexist in Type II pneumocytes, ileal absorptive enterocytes, and nasal goblet secretory cells [1]. Type II pneumocytes are the only cells that produce and secrete pulmonary surfactant (PS), which prevents alveoli from collapsing by reducing the surface tension of the alveolar liquid layer [2]. Iwasawa et al. reported that ultra-high-resolution X-ray CT images in coronavirus disease of 2019 (COVID-19) pneumonia showed volume loss of the lung parenchyma because of alveolar collapses [3]. The alveolar collapse causes mechanical injury of capillaries located in the alveolar wall and allows the virus to bind ACE2 on the surface of endothelial cells in the alveolar capillary.

Acute respiratory distress syndrome (ARDS) was first reported in 1967 by Ashbaugh et al., who inferred that its essential pathology was alveolar collapse caused by PS dysfunction and likely infant respiratory distress syndrome [4]. Currently, ARDS is defined as non-cardiogenic pulmonary edema with vascular hyper-permeability; the role of PS dysfunction is regarded as subtle [5]. This conceptual change may be partly because of the clinical ineffectiveness of PS instillation therapy for adults. Several clinical trials indicated that PS instillation therapy failed in reducing mortality in spite of improvements in blood oxygenation [6, 7]. However, there is a possibility that this ineffectiveness is caused by insufficient delivery owing to the significant difference in size and structure between newborn and adult lungs [7, 8]. Additionally, several researchers have reconsidered classical concepts of interstitial pneumonia and stated that alveolar collapse is the predominant mechanism of diffuse alveolar damage, which is a typical pathologic change in ARDS, acute interstitial pneumonia, and acute exacerbation of idiopathic pulmonary fibrosis [9, 10]. Steffen et al. recently demonstrated the experimental therapeutic effects of surfactant replacement for acute lung injury in a rat bleomycin model [11].

Therefore, it is apparent that the loss of PS because of the infection of Type II pneumocytes causes diffuse alveolar damage in COVID-19. Gattinoni et al. reported that the respiratory system compliance is almost normal in the initial stages of COVID-19 pneumonia [12]. It may be because regional low distensibility due to alveolar collapses is compensated by over-inflation of the surrounding intact lung parenchyma. Hence, supplying PS to the diseased alveolar regions to recover alveolar collapses is one of the most important strategies for ARDS by COVID-19. Filoshe et al. performed simulations of PS instillation and concluded that current PS delivery methods should be improved [13]. The airway models in their study were a combination of cylinders of varying calibers representative of the cascading lung bifurcations without a continuous surface. In this study, we simulated PS instillation in a realistic three-dimensional (3D) human airway model [14] using computational fluid dynamics (CFD).

## Methods

**Fig. 1:**
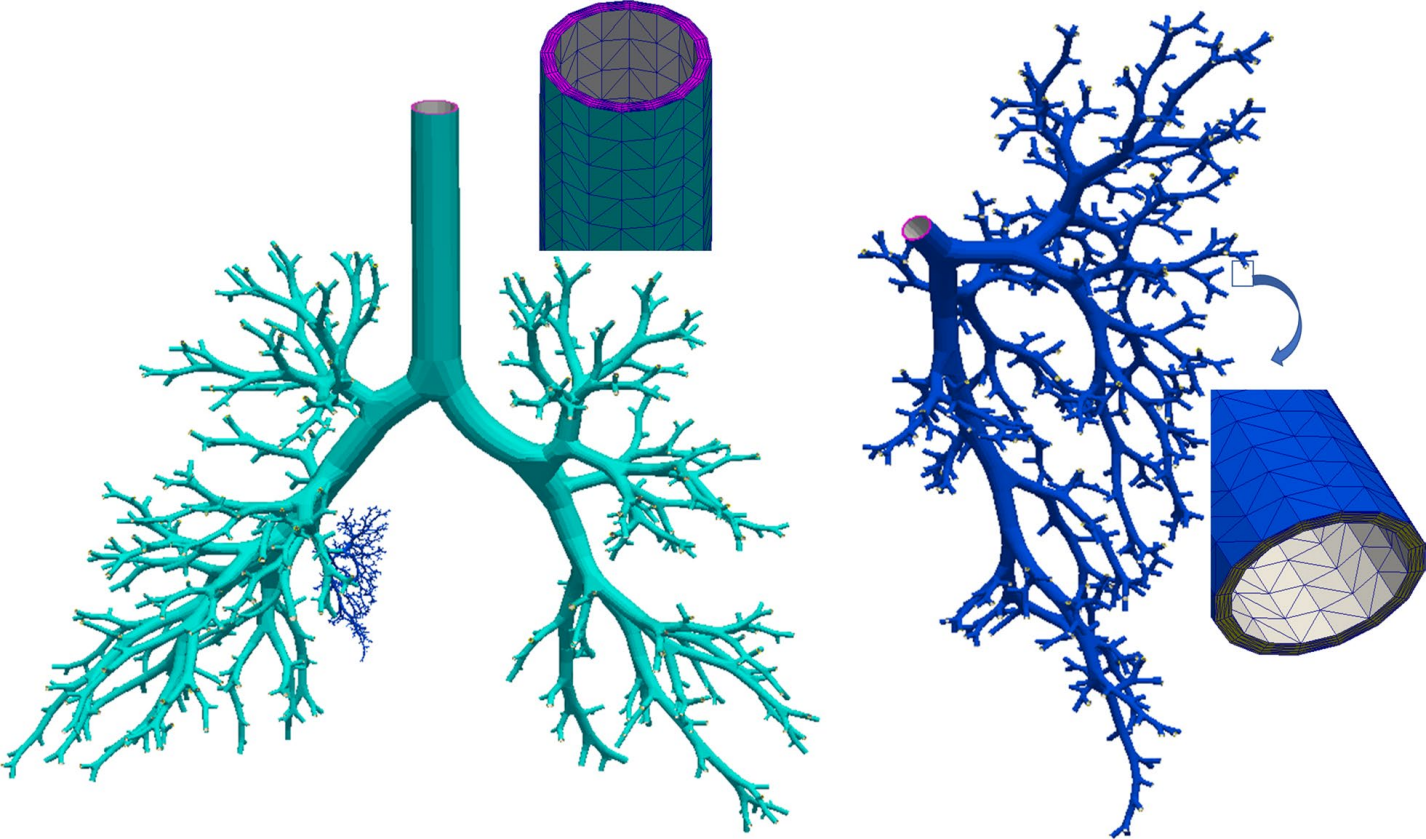
Three-dimensional human airway tree model. The entire model (light blue) and subsegmental model (dark blue). Pink = proximal end surface of liquid layer; yellow = distal end surfaces of liquid layer; white = surface of liquid layer facing air

Fluid flow in the liquid layer covering the airway wall driven by PS instillation was computed using a CFD solver (acuSolve, Altair Engineering, USA). Because applying CFD to the entire airway tree from the trachea to respiratory bronchioles requires substantial computer resources, two types of 3D human airway models were prepared. One was from the trachea to the lobular bronchi (Fig. 1, light blue part), and the other was from a subsegmental bronchus to respiratory bronchioles corresponding to the posterior subsegment of the basal medial segment in the right lung (rtS7a, Fig. 1, dark blue part), which is the smallest subsegment in the human lung. The number of end segments in the entire model was 531, and their mean diameter was 1.92 mm (total lung capacity). The subsegmental model had 511 end segments corresponding to respiratory bronchioles with a mean diameter of 0.35 mm. Because the algorithm for the 3D airway tree model was devised to assign the air-supplying region of lung parenchyma for each segment [15], the parenchymal volume in the subsegmental model was automatically recognized as 1.5 % of the entire lung volume. The entire model consisted of 1,929,371 nodes and 8,531,760 finite elements (tetras), whereas the subsegmental model consisted of 1,802,620 nodes and 7,960,320 tetras.

According to the simulated results by Filoshe et al. [13], the thickness of the liquid layer was 14 % of the inner radius of the airway segment. The liquid layer was assumed to be replaced by an instilled PS with the same thickness. The fluid surface facing the airway wall (green or blue parts in Fig. 1) were set as non-slip condition (velocity is zero for all directions), and the fluid surface facing air (white parts in Fig. 1) were set as slip (velocity is zero for only the normal direction) condition. The flow rate of the instilled PS was assigned a constant value, which was determined by the total amount and instillation time in clinical use according to Filoshe et al. [13]. For simplicity, the PS instillation was assumed to be evenly distributed at the proximal end surface of the liquid layer (pink part in Fig. 1). The assigned flow rate of the subsegmental model was proportional to the volume of the subsegment. The PS concentration of the liquid layer during instillation was computed by solving the advective–diffusion equation at each time step (0.01 s). The viscosity of the instilled PS was set as 30 cP [13]. The diffusivity of PS to existing liquid layer was assigned at 10^− 10^ m^2^/s. The densities of the liquid layer and PS were assigned 1.0 kg/L.

## Results

**Fig. 2:**
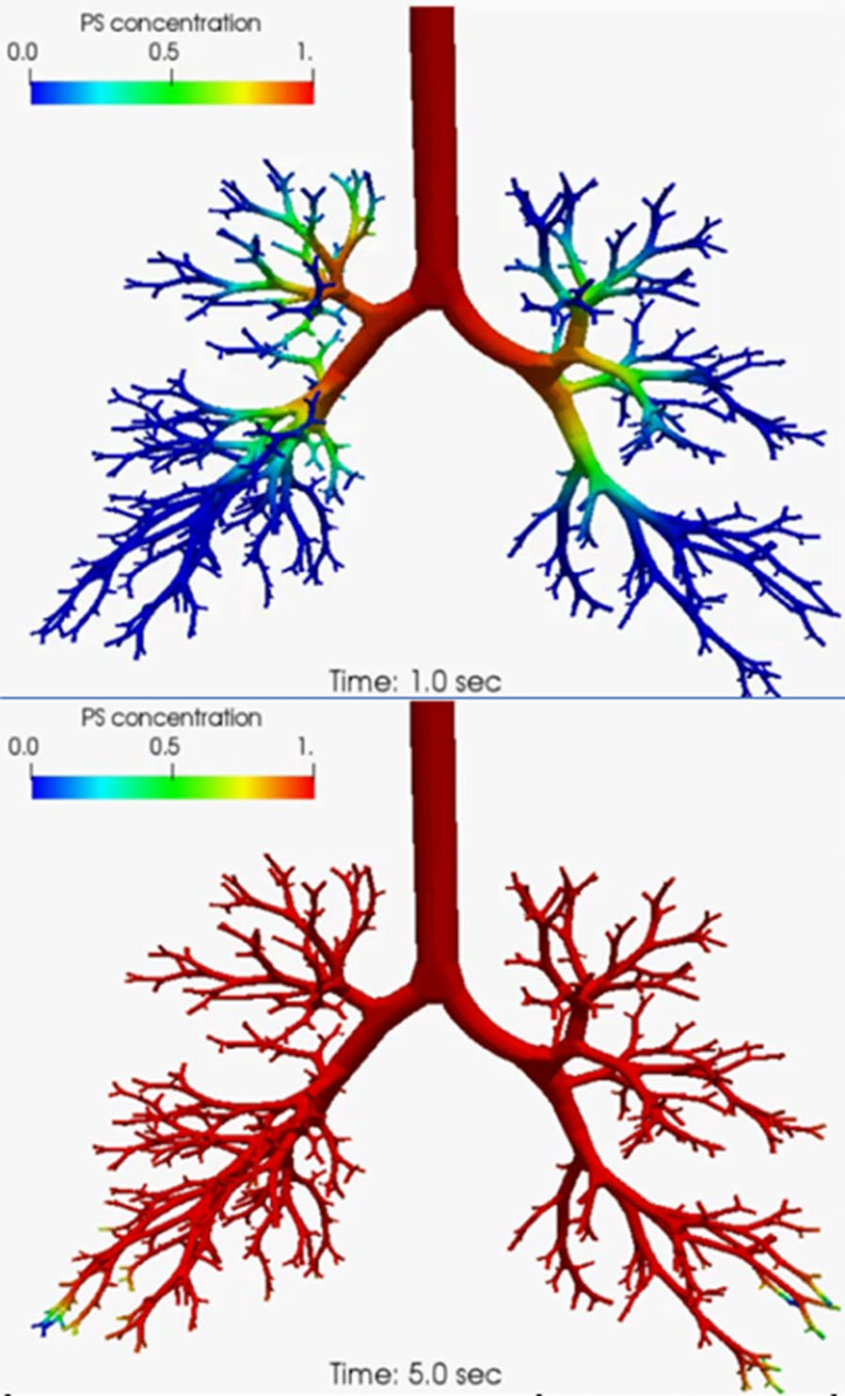
PS concentration distributions in the entire model at one second after (upper) and five seconds after (lower)

**Fig. 3:**
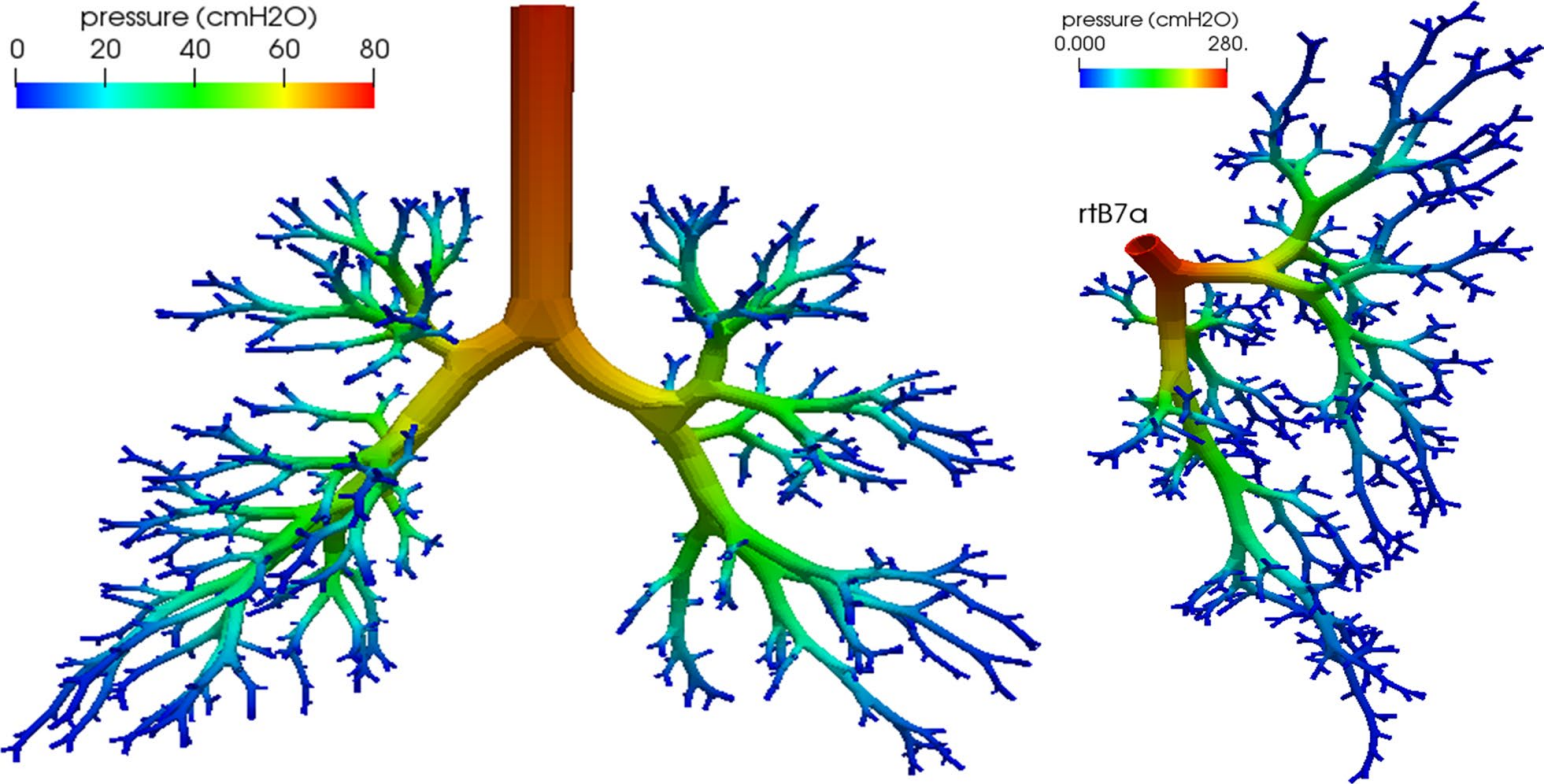
Pressure distribution during PS instillation in the entire model (left) and subsegmental model (right). Note that the color scales are different

Figure 2 show PS concentration distributions on the free surface of the liquid layer of the entire model. There is an apparent gradient of PS concentration approximately proportional to the path length from the top at one sec after the beginning of instillation. Five seconds after, PS with 100 % concentration is drained through almost all terminal segments except the most distal regions in the lower lobes. In the sub-segmental model, the arrival time of PS to the terminal segments was nearly equal to that in the entire model. However, the driving pressure to realize a given flow rate was quite high. Figure 3 shows the pressure distribution in the liquid layer during instillation. The driving pressure at the inlet was calculated as 76 cm H_2_O in the entire model and 273 cm H_2_O in the subsegmental model. The extremely high driving pressure in the subsegmental model was caused by the smaller cross-sectional area of the liquid layer. Because the pressure at rtB7a in the entire model was 32 cm H_2_O, the total pressure between the trachea and alveoli was approximately 317 cm H_2_O (= 76 – 32 + 273).

## Discussion

Our simulation has revealed that the driving pressure for PS instillation to the alveolar region is much larger than that to the large bronchi. The smaller the airway is, the larger the pressure drop is due to the liquid viscosity. Because the conventional method of PS instillation uses an endotracheal tube, the driving pressure of the PS to the alveoli is equal to the difference between the inspiratory and end-expiratory pressures of a ventilator [6, 7], which is approximately one-twentieth of the computed value in our simulation. This suggests that almost all the PS may not reach the alveolar regions but move to and from within the airway according to the change in ventilator pressure, or occlude the intra-airway space by plug formation, which causes regional atelectasis. This may be the reason for the ineffectiveness of conventional PS instillation therapy for adults. The effectiveness of conventional intra-tracheal PS instillation in newborns may be because the relative airway size to the entire lung is significantly larger in newborns owing to immature alveoli, especially in premature newborns. Hence, the required driving pressure for PS instillation may be significantly smaller in premature newborns than that in adults.

The instillation of the subsegmental bronchus can be performed by a consecutive wedge pressure of approximately 300 cm H_2_O, similar to the selective alveolo-bronchography (SAB) technique [16] where contrast medium is supplied by wedge instillation from the segmental bronchi. Although SAB has not been applied for three decades owing to the emergence of high-resolution CT imaging, its methodology is well established. Because damaged alveolar regions can be identified by CT images, selective and simultaneous instillations of PS and anti-viral agents are executable under bronchoscopic observation before the onset of ARDS. It may also be useful for preventing secondary lung fibrosis and viral binding to ACE2 in capillary endothelial cells which may cause systemic vascular disorders.

There are several limitations in our simulation: experimental data on the liquid layer thickness are not available and the displacement of the free surface is neglected. If the assumed value of the thickness is different, computed value of the driving pressure becomes different. Recently, five cases of intra-tracheal PS instillation therapy under mechanical ventilation for severe ARDS due to COVID-19 pneumonia were reported, and there was a positive outcome in four [17]. They used a three-way tap connected to the closed-loop suction catheter inserted into the endotracheal tube with the distal hole approximately 1 cm above the carina, and a half of the volume was administered while the patient was in the right lateral decubitus position, with the remaining dose given in the opposite lateral position 5 min apart. Such a delicate method may be one of the reasons of the positive outcome, although the number of reported cases is too small to evaluate the effect of surfactant instillation.

## Conclusions

Based on the CFD simulation results, we propose selective wedge instillations of PS from subsegmental bronchi under bronchoscopic observation for COVID-19 pneumonia with complete infection protection. It will be also useful for preventing secondary lung fibrosis and other complications. Practical strategies for PS instillation including timing, dosage, and the type of surfactant, should be carefully considered, although these issues are beyond the ability of this simulation study.

## Data Availability

The datasets used and/or analyzed during the current study are available from the corresponding author on reasonable request.

## Abbreviations

COVID-19: Coronavirus disease of 2019
PS: Pulmonary surfactant
ARDS: Acute respiratory distress syndrome
ACE2: Angiotensin converting enzyme 2
CFD: Computational fluid dynamics
SAB: Selective alveolo-bronchograph
